# Fit Entropy-Based Dynamic Communication Resource Slicing-Optimization Method in Smart Distribution Grids on the Medium–Low-Voltage Side

**DOI:** 10.3390/s24175522

**Published:** 2024-08-26

**Authors:** Haiming Li, Xiaorong Zhu

**Affiliations:** College of Telecommunications and Information Engineering, Nanjing University of Posts and Telecommunications, Nanjing 210003, China

**Keywords:** power communication network, three-domain model, hypergraph, service function chain, fit entropy, utility

## Abstract

With the rapid development of new energy and smart technology, the demand for inter-device communication in medium–low-voltage smart distribution grids has sharply increased, leading to a surge in the variety and quantity of communication services. To meet the needs of diverse and massive communication services, deploying service function chains to flexibly combine virtual resources has become crucial. This paper proposes an optimization method based on fit entropy and network utility to address the limited communication network resources in medium–low-voltage smart distribution grids. This was conducted by modeling the distribution grid as a three-domain model consisting of a service domain, a logical domain, and a physical domain and transforming it into a hierarchical bipartite hypergraph-matching problem, which is a complex combinatorial optimization problem. This paper introduces two matching optimization algorithms: “business domain–logic domain–physical domain integration” and “service domain–logic domain, logic domain–physical domain two-stage”, which effectively address this problem based on fit entropy and utility. The simulation results demonstrate that these algorithms significantly improve service success rates and resource utilization, enhancing overall network utility.

## 1. Introduction

With the rapid development of new energy and the widespread application of intelligent technology, the power system is undergoing a transformation from a traditional structure to highly automated and intelligent operations. Power terminals are also rapidly moving towards full connectivity and real-time interaction. The number of terminal devices in the communication network of medium–low-voltage distribution grids will significantly increase, leading to a manifold growth in service requests. In this process, with the widespread use of technologies such as distributed energy and electric vehicles, various new types of services have emerged in the communication network of smart distribution grids on the medium–low-voltage side. These services require data from sensors of different scales and types in the network at different intervals, demanding high service capabilities from the network. For example, in the case of fee-control collection services, ESAM (Embedded Security Module) information from all energy meters in the entire area needs to be collected every day, authenticated, and controlled instantly, which requires high real-time interaction and reliability from the network.

To meet the differentiated performance requirements of diverse services for network resources, flexible adjustments in resource allocation are urgently needed to cope with changing business scenarios. Traditional power communication networks mainly use a combination of hardware and software in the adjustment process, which is costly and requires professional engineers to configure the network. However, the communication network of smart distribution grids on the medium–low-voltage side, using network function virtualization technology, achieves the separation of software and hardware. It allows network functions to run in standard infrastructure rather than in custom hardware, and virtual network functions have the same processing capabilities as hardware network functions, greatly increasing the flexibility of network management.

Although the adoption of network-function virtualization technology in the communication network of smart distribution grids on the medium–low-voltage side brings many benefits, it still faces some key challenges. Specifically, in the context of limited network resources, we must establish how to deploy multiple service function chains in the network to meet the QoS requirements of different services as much as possible and improve the resource utilization of the network. In this context, there is an urgent need for a comprehensive assessment method for the communication network of smart distribution grids on the medium–low-voltage side that considers various factors such as business characteristics, network service capabilities, and cost-effectiveness to evaluate the degree of alignment between the network and the business and optimize it.

Current communication network evaluation methods predominantly focus on assessing network topology and performance parameters, which have certain limitations. Some studies, based on complex network theory, utilize features like high clustering coefficients to compute the end-to-end reliability of networks [1]. Other research combines mobile internet technologies to construct artificial intelligence models by monitoring various types of communication devices, thereby evaluating communication systems [2]. Additionally, there are studies proposing multi-index critical node-identification algorithms based on the centrality of structural holes, analyzing network vulnerabilities from both network and business layers [3]. On another front, a network performance-evaluation model system has been designed for smart-grid demand analysis, assessing metrics such as communication bandwidth, speed, and device congestion [4]. Researchers have also introduced reliability assessment methods for distributed renewable energy-based power communication networks, analyzing their reliability through specific indicators like fault rates [5]. Furthermore, based on the operational characteristics of power communication, certain studies use link transmission traffic as link weights, constructing network-link diversity matrices and defining node importance and the structural entropy of power backbone communication networks to analyze network resilience using structural entropy as an indicator [6].

With the emergence of network virtualization technology, existing hardware networks are transformed into software-based networks. The completion of business tasks in the network can be seen as the process of allocating physical network resources to virtual network requests (VNRs), referred to as the virtual-network mapping problem. Existing methods primarily focus on either business priority or network priority. Business-oriented approaches typically address specific metrics like latency sensitivity and reliability [7,8], while network-oriented approaches focus on aspects like energy consumption and load balancing [9,10,11,12,13]. Ref. [14] solves the model through linear programming to obtain an initial mapping scheme. If it is infeasible, it reselects the mapping path and verifies it in the dual model, thereby improving the mapping success rate and reducing the execution time of virtual network mapping. Ref. [15] proposes using queueing theory to achieve VNF resource allocation, but this method is only applicable to fixed resource allocation. Moreover, this method requires specialized probes to monitor different queue sizes in VNFs, which reduces its practicality.

These studies have made some progress in different directions, but in smart-distribution-grid communication networks and future networks, the types and demands of services are complex and diverse. It is not sound to solely consider either business or network aspects. Therefore, this paper focuses on the overall architecture of the communication network in smart distribution grids on the medium–low-voltage side. Based on the business requirements in the network, a resource–business alignment evaluation and optimization method are established to improve network resource utilization while meeting business QoS. The main contributions are as follows:

To characterize the complex and diverse relationships between different combinations of business attributes, service function attributes, and device performance attributes in smart distribution grids on the medium–low-voltage side, a three-domain model consisting of a service domain, a logical domain, and a physical domain is designed, based on the communication network architecture.

To evaluate the degree of alignment between business and network service capabilities within the network, fit entropy is defined based on the probability distributions of business demand in terms of latency, reliability, and accuracy, as well as the probability distributions of service function chains that the network can provide across these three dimensions.

To assess the network’s resource utilization and service capabilities, network utility is defined in conjunction with fit entropy and network service costs. An optimization problem is proposed based on maximizing network utility, aiming to meet business requirements and optimize network resource utilization without exceeding network load.

To describe the multi-to-multi high-order matching relationships between business, service function chains, and physical components in the network, the three-domain model of the communication network in smart distribution grids on the medium–low-voltage side is described based on hypergraph theory. The matching process of the physical component of the business–service function chain is transformed into a hierarchical bipartite hypergraph-matching problem. Two matching optimization algorithms, namely “service domain–logical domain–physical domain integration” and “service domain–logical domain, logical domain–physical domain two-stage,” are proposed and verified through simulation.

## 2. System Model

### 2.1. Communication Network Architecture for Smart Distribution Grids on the Medium–Low-Voltage Side

**Figure 1:**
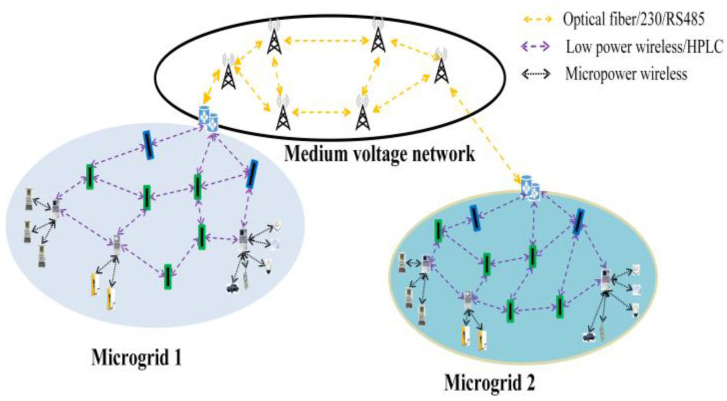
Communication network for smart distribution grids on the medium–low-voltage side.

This paper considers the communication network scenario for smart distribution grids on the medium–low-voltage side, as depicted in Figure 1. The network scenario mainly consists of multiple microgrids and network exchange centers. Microgrids serve as the basic units, forming a multi-layered, interconnected, large-scale meshed information network that is flexibly interconnected on demand. This network supports massive heterogeneous terminals for seamless access and meets the requirements for micro-scheduling. Within each microgrid, there is a high degree of autonomy, allowing for interaction with the distribution grid and information exchange. This setup addresses the challenges posed by the large-scale integration of new energy sources and new types of loads, enabling plug-and-play functionality.

As network demands increase, a large number of network middleware components are added to the network. Additionally, the diversification of services and requirements necessitates the continuous updating of physical equipment in the power network. However, as these devices are typically designed and implemented by different manufacturers, there are significant differences in hardware architecture, leading to compatibility challenges. This complexity in network management results in high capital expenditure and operational costs.

To address these issues, this paper assumes the power network consists of physical, logical, and service domains. The purpose of the logical domain is to abstract the functionality of physical devices and decouple them from the physical domain, forming a service function network. This separation enables the service domain to be indirectly matched with the physical domain, enhancing network flexibility and reducing both capital expenditure and operational costs.

**Figure 2:**
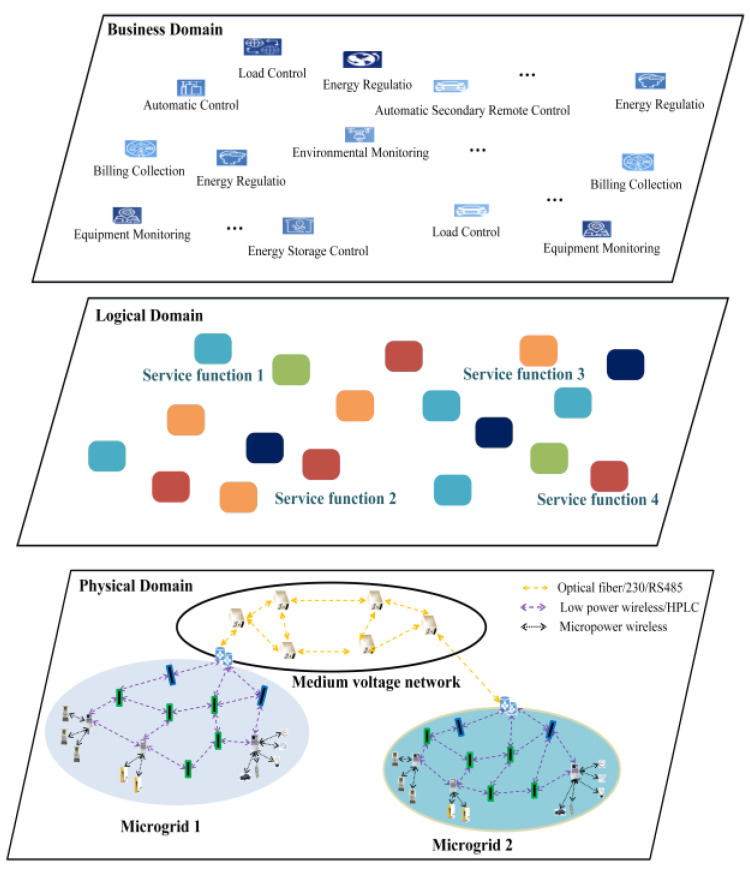
The three-domain model of communication network for smart distribution grids on the medium–low-voltage side.

The scenario is modeled as a three-domain model, as shown in Figure 2, which includes the service domain, logical domain, and physical domain. The definitions and representations of each domain in the three-domain model are introduced separately.

Physical Domain: The physical domain in the communication network of smart distribution grids on the medium–low-voltage side abstracts and models the physical devices that provide basic resources and operate network functions, including access networks, transport networks, core networks, and other physical equipment. It is defined as $W=\left\{w_{1},w_{2},\ldots w_{k},\ldots \right\}$.

Here, $w_{k}$ represents the physical component k, and $w_{k}=\left\{type_{k}^{w},res_{k}^{w},pos_{k}^{w}\right\}$ is used to describe the specific attributes of this component. Here, $type_{k}^{w}$ represents the type of physical component k, such as microgrid marketing equipment, microgrid distribution equipment, and medium-voltage grid transmission equipment. $res_{k}$ represents the location information of physical component k. $res_{k}^{w}$ represents the amount of resources available for physical component k, including computational resources $c_{k}^{w}$, storage resources $r_{k}^{w}$, spectrum resources $f_{k}^{w}$, and power resources $p_{k}^{w}$, represented as $res_{k}^{w}=\left\{c_{k}^{w},r_{k}^{w},f_{k}^{w},p_{k}^{w}\right\}$.

Logical Domain: The logical domain divides the existing communication network of smart distribution grids on the medium–low-voltage side into logical segments. It abstracts the functionality of different physical components and splits or combines them to form a set of service functions. For business operations with varying requirements, independent service functions are combined and scheduled to form isolated service function chains with negotiable and customizable management capabilities. The logical domain is defined as $L=\left\{l_{1},l_{2},\ldots l_{k},\ldots \right\}$.

Here, $l_{k}$ represents the service function k, and $l_{k}=\left\{type_{k}^{l},res_{k}^{l},perf_{k}^{l}\right\}$ is used to describe the specific attributes of this service function. $type_{k}^{l}$ represents the category of service function k, such as a firewall, routing forwarding, a gateway, etc. $res_{k}^{l}$ represents the amount of resources consumed by service function k per unit traffic, including computational resources $c_{k}^{l}$, storage resources $r_{k}^{l}$, spectrum resources $f_{k}^{l}$, and power resources $p_{k}^{l}$, represented as $res_{k}^{l}=\left\{c_{k}^{l},r_{k}^{l},f_{k}^{l},p_{k}^{l}\right\}$. $pref_{k}^{l}$ represents the performance indicators of service function k for processing per unit traffic, including delay $delay_{k}^{l}$, reliability $rel_{k}^{l}$, and precision $prec_{k}^{l}$, represented as $pref_{k}^{l}=\left\{delay_{k}^{l},rel_{k}^{l},prec_{k}^{l}\right\}$.

Service Domain: The service domain abstractly models various business scenarios generated in the communication network of smart distribution grids on the medium–low-voltage side. Different technological combinations are adopted based on the differentiated requirements of internal network services, and corresponding service function chains are customized on demand in the logical domain. Initially, this paper predefines the types and specific requirements of various businesses based on the diversity of business categories and demands. Let S represent the collection of all businesses in the service domain, defined as $S=\left\{s_{1},s_{2},\ldots ,s_{k},\ldots \right\}$.

Here, $s_{k}$ represents business k, and $s_{k}=\left\{type_{k}^{s},Ds_{k},R_{k}\right\}$ is used to describe the specific requirements of this business. $type_{k}^{s}$ represents the type of business k, $Ds_{k}$ represents the data volume size of business k, and $R_{k}$ represents the description of the types and levels of requirements for business k. Business requirements mainly consider delay, precision, and reliability.

In the communication network of smart distribution grids on the medium–low-voltage side, the business exhibits a certain level of periodicity. The requirement levels of various businesses are statistically analyzed over a period of time. Businesses are divided into different requirement levels based on their demands, described as $R_{k}=\left\{delay_{k}^{s},rel_{k}^{s},prec_{k}^{s}\right\}$, representing the levels of delay, reliability, and precision requirements. The corresponding table is as follows:

### 2.2. Service and Resource Matching Process

**Figure 3:**
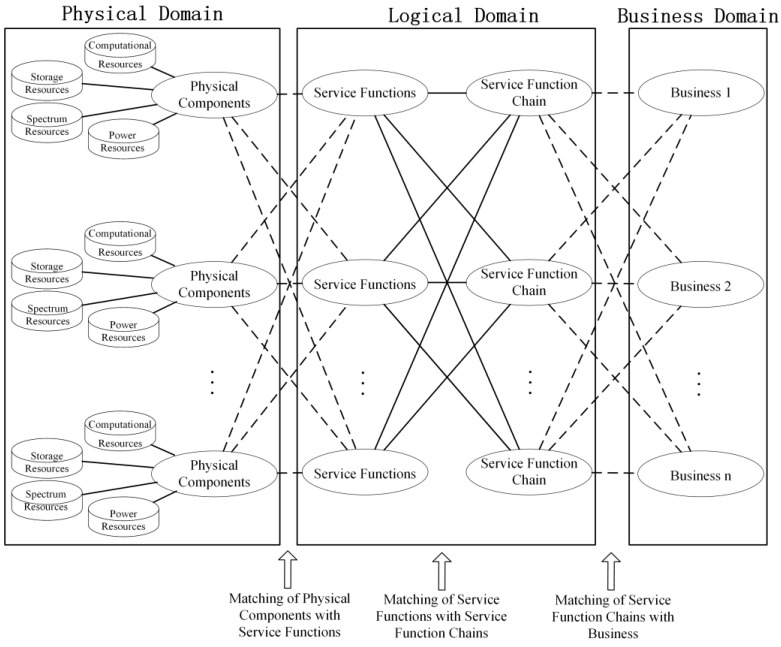
Illustration of three-domain matching.

To evaluate the alignment between the communication network of smart distribution grids on the medium–low-voltage side and the internal network services, this paper views the execution process of network services as a three-tier matching process, as illustrated in Figure 3. Firstly, based on the requirements of business in the service domain, the service function chain (SFC) requests are generated. Then, according to all the types of service functions in the requests, one or more service function chains that meet their requirements are searched for in the logical domain. Subsequently, under the condition of meeting resource requirements, the service functions in the service function chain are matched and mapped to the physical devices in the physical domain. Since the micro-services are formed by abstracting the functionality of physical devices, one physical device can correspond to multiple micro-services, and similarly, one micro-service can match multiple physical devices.

Define $R^{S\to L_{SFC}}$ as the mapping probability matrix from the service domain to the logical-domain service function chains. The matrix size is $N_{S}\times N_{SFC}$, where $N_{SFC}$ represents the number of service function chains. Matrix element $R_{ij}^{S\to L_{SFC}}$ indicates the probability of mapping the business $s_{i}$ to the service function chain $SFC_{j}$, denoted as

(1) $$R^{S\to L_{SFC}}=\left\{\left.R_{ij}^{S\to L_{SFC}}=p_{ij}\right|\sum_{j=1}^{N_{SFC}}p_{ij}=1,\;\forall i\in \left[1,N_{S}\right]\right\}$$

$SFCG_{i}$ is defined as the set of service function chains in the logical domain that have a probability mapping with the business $s_{i}$, denoted as

(2) $$SFCG_{i}=\left\{\left.\overset{N_{SFC}}{\underset{j=1}{\cup }}SFC_{j}\right|R_{ij}^{S\to L}\neq 0,\forall i\in \left[1,N_{S}\right]\right\}$$

The relationship between service function chains and service functions in the logical domain is represented by the association matrix $R^{L_{SFC}\to L}\in {\left\{0,1\right\}}^{N_{SFC}\times N_{L}}$. Then, the mapping probability matrix $R^{S\to L}$ from the service domain to the service functions in the logical domain can be obtained through the row normalization of $R^{S\to L_{SFC}}\times R^{L_{SFC}\to L}$.

Similarly, define $R^{L\to W}$ as the mapping probability matrix from the logical domain to the physical domain. The size of the matrix is $N_{L}\times N_{W}$, where each element represents the probability of service function $l_{j}$ mapping to physical component $w_{k}$, denoted as

(3) $$R^{L\to K}=\left\{\left.R_{jk}^{L\to K}=p_{jk}\right|\sum_{j=1}^{N_{W}}p_{jk}=1,\forall j\in \left[1,N_{L}\right]\right\}$$

For the business side, each business aims to have service function chains that meet its business requirements. Due to the stochastic and multidimensional nature of business demands, selecting matches based on a single criterion is not comprehensive. Therefore, this paper evaluates the alignment between service function chains and business using the concept of fit entropy. The following introduces the definition of fit entropy.

### 2.3. Definition of Fit Entropy

To effectively evaluate the alignment between service function chains and business demands, we present and explore the concept of fit entropy. Fit entropy is particularly suitable for this context because it is designed to match the random distributions based on cross-entropy.

Based on the description $R_{k}=\left\{delay_{k}^{s},rel_{k}^{s},prec_{k}^{s}\right\}$ of the time delay requirement, reliability requirement, and the precision requirement of business $s_{k}$, micro-service functions in the logical domain form service function chains according to business needs. Due to the diversity in the performance of individual service functions, there is no unique service function chain that meets business requirements. The set of service function chains that satisfy business requirements is referred to as a service-function-chain group.

Suppose one of the service function chains is $SFC_{i}^{k}=\left\{SF_{1}^{k},SF_{2}^{k},\ldots ,SF_{N_{i,k}}^{k}\right\}$, where $N_{i,k}$ is the number of service functions in the service function chain matched to business $s_{k}$. Define the delay service level of service function chain $SFC_{i}^{k}$ as $delay_{i}^{k}=f_{delay}\left(Ds_{k}\prod_{j=1}^{N_{i,k}}delay_{j}^{l}\right)$, where $f_{delay}$ maps values to delay levels. Similarly, the reliability service level $rel_{i}^{k}=f_{rel}\left(Ds_{k}\prod_{j=1}^{N_{i,k}}rel_{j}^{l}\right)$ and precision service level $prec_{i}^{k}=f_{prec}\left(Ds_{k}\prod_{j=1}^{N_{i,k}}prec_{j}^{l}\right)$ can be defined.

Suppose there are $N_{k}$ service function chains matched with business $s_{k}$, and the probability group of matches $P_{k}=\left[p_{1},p_{2},\ldots p_{N_{k}}\right]$ represents the probability of each service function chain being matched. Due to the uncertainty of the matched service function chains, the provided service levels are also uncertain. Taking delay capability as an example, the probability group of the capability of the matched service-function-chain group with the business can be represented as

(4) $$delay_{e}=\left\{P_{e}^{1},P_{e}^{2}\ldots P_{e}^{k}\ldots P_{e}^{N_{delay}}\right\}$$

where $P_{e}^{1}+P_{e}^{2}+\ldots +P_{e}^{N_{delay}}=1$, $N_{delay}$ represents the maximum number of levels of delay capability, and $P_{e}^{k}$ is the probability of the delay capability service level being k. This is derived from the expectation of the capability levels of each service function chain in the group according to the matching probability.

Similarly, reliable capability and precision capability can be specifically expressed. Meanwhile, define conformity entropy to measure the degree of conformity between the service capability provided by the service-function-chain group and the service capability required by the business, represented as E. Due to the multidimensionality of service capability, conformity entropy is divided into multiple parts. If the business’s delay statistical requirement is $delay=\left\{P^{1},P^{2}\ldots P^{k}\ldots P^{N_{b}}\right\}$, then the expression of delay conformity entropy is defined as

(5) $$E^{delay}=-\sum_{i=1}^{N_{delay}}P^{i}\log\left(P_{e}^{i}\right)mP_{e}^{i}\neq 0$$

Similarly, expressions for reliability conformity entropy $E^{rel}$ and precision conformity entropy $E^{prec}$ can be defined. The conformity entropy is composed of the weighted sum of these parts, represented as

(6) $$E=\lambda_{delay}E^{delay}+\lambda_{rel}E^{rel}+\lambda_{prec}E^{prec}$$

Here, $\lambda_{delay}$, $\lambda_{rel}$, and $\lambda_{prec}$ are the weighting coefficients for the delay conformity entropy $E^{delay}$, reliability conformity entropy $E^{rel}$, and precision conformity entropy $E^{prec}$, respectively.

Based on the conformity entropy, we can represent the degree of conformity between the power network and the internal business requirements—that is, the degree of matching between the network and the business. However, the conformity entropy only reflects the business requirements and does not consider the costs incurred in meeting the business requirements in the network. Blindly optimizing conformity entropy would significantly increase network costs. Therefore, this paper defines business utility to simultaneously weigh both aspects.

### 2.4. Business Utility Definition

In economics, utility refers to the measure of satisfaction or fulfillment that consumers derive from the consumption of goods or services, as well as from the enjoyment of leisure activities. Economists use it to explain how rational consumers allocate their limited resources to maximize satisfaction by choosing goods or services that provide the greatest benefit.

In this study, the network utility value depends on the degree of alignment between the capabilities of the service-function-chain group matched with the business in the network and its requirements, as well as the service costs required to implement these service functions. The degree of alignment between the business and its matched service-function-chain group is represented by the conformity entropy. Next, the expressions for service costs and network utility values will be introduced.

Service cost refers to the total resources consumed by the service-function-chain group matched with the business. The types of resources include computing resources, storage resources, spectrum resources, and power resources. Taking service function $l_{k}$ as an example, the cost of using service function $l_{k}$ is related to the size of the data Ds and the cost of using various types of resources. It is defined as

(7) $${\mathrm{cost}}_{k}=Ds\left(c_{k}+r_{k}+f_{k}+p_{k}\right)$$

In this paper, network utility is related to the cost of business service and the fit entropy E. When the fit entropy E is smaller, the cost of business service is also smaller, resulting in a larger system utility value U. Conversely, when the fit entropy E is larger, the cost of business service is also larger, leading to a smaller system utility value U. Based on these properties, a specific form of network utility function can be derived as

(8) $$U\left(E,\mathrm{cost}\right)=1-e^{-\chi_{1}\cdot E^{-\chi_{2}}\cdot \cos t^{-\chi_{3}}}$$

where $\chi_{1},\chi_{2},\chi_{3}$ are all positive constants.

## 3. Optimization of the Matching between the Service Domain, Logical Domain and Physical Domain Based on Utility

### 3.1. Problem Formulation

The service domain must first select multiple service function chains in the logical domain for matching. These chains are ordered or partially ordered sequences composed of service functions, and data packets, frames, or streams must adhere to their specified sequential constraints. The order of service function chains may not be linear and can allow for multiple branches.

The optimization objective is to maximize the utility of the communication network in the low- and medium-voltage intelligent distribution grid, leading to the following optimization problem formulation:

$$\begin{array}{ll} s.t. & C1:\sum_{i=1}^{N_{Y}}\sum_{j=1}^{N_{L}}\sum_{k=1}^{N_{W}}R_{ij}^{S\to L}R_{jk}^{L\to W}c_{j}^{l}\leq c_{k}^{w}\\ & C2:\sum_{i=1}^{N_{Y}}\sum_{j=1}^{N_{L}}\sum_{k=1}^{N_{W}}R_{ij}^{S\to L}R_{jk}^{L\to W}r_{j}^{l}\leq r_{k}^{w}\\ & C3:\sum_{i=1}^{N_{Y}}\sum_{j=1}^{N_{L}}\sum_{k=1}^{N_{W}}R_{ij}^{S\to L}R_{jk}^{L\to W}f_{j}^{l}\leq f_{k}^{w}\\ & C4:\sum_{i=1}^{N_{Y}}\sum_{j=1}^{N_{L}}\sum_{k=1}^{N_{W}}R_{ij}^{S\to L}R_{jk}^{L\to W}p_{j}^{l}\leq p_{k}^{w}\\ & C5:\sum_{j=1}^{N_{L}}R_{ij}^{S\to L}=1,\forall i\in \left[1,N_{Y}\right]\\ & C6:\sum_{k=1}^{N_{W}}R_{jk}^{L\to W}=1,\forall j\in \left[1,N_{L}\right]\\ & C7:\mathrm{SevL}\left(k\right)\geq R_{i}(k),\mathrm{SevL}\in SFCG_{i},1\leq k\leq 3 \end{array}$$

Here, $N_{Y}$ represents the number of businesses in the service domain, $N_{L}$ represents the number of service functions in the logical domain, $N_{W}$ represents the number of physical components in the physical domain, and $E_{i}$ represents the entropy of fit between the business and its matched service function chain. $c_{j}$ represents the computational resource required by service function j. Similarly, $r_{j}$ represents the storage resource, $f_{j}$ represents the required frequency resource, and $p_{j}$ represents the required power resource. $c_{k}^{\max}$ represents the maximum computational resource that physical component k can provide. Similarly, $r_{k}^{\max}$ represents the maximum storage resource, $f_{k}^{\max}$ represents the maximum frequency resource, and $p_{k}^{\max}$ represents the maximum power resource. $SevL\left(k\right)$ represents the kth dimension capacity level of the service function chain, while $R_{i}\left(k\right)$ represents the kth dimension demand of service $s_{i}$. $SFCG_{i}$ represents the service-function-chain group matched with service $s_{i}$. Constraints C1,C2,C3,C4, respectively, represent that the required computation, storage, frequency, and power resources of the service function chain cannot exceed the maximum values of the mapped physical devices. C5,C6 represent normalization limits for mapping probabilities, while C7 indicates that the service level of the matched service function chain must be greater than the demand level of the business.

The optimization problem of tri-domain matching essentially involves matching between service domains and logical-domain service function chains, as well as matching between logical-domain service functions and physical domain components. It can be viewed as a graph matching problem. Since graph matching problems themselves are combinatorial optimization problems, it is difficult to find their global optimal values. Typically, constraints in the optimization of graph matching problems are relaxed to obtain approximate optimal solutions. Current mainstream graph-matching algorithms roughly fall into spectral methods, continuous relaxation optimization methods, probability-based methods, and learning-based methods. This paper mainly focuses on hypergraph matching methods.

### 3.2. The Matching Mechanism between the Service Domain, Logic Domain, and Physical Domain

The three-domain utility-optimization matching problem involves matching businesses with service function chains and service functions with physical components. The nodes in the three domains are mutually exclusive and can be seen as two bipartite graph-matching problems. However, because businesses are matched with service function chains in the logic domain, which involves a one-to-many relationship, it is not straightforward to describe using a simple bipartite graph. A hypergraph, on the other hand, is a generalized form of a graph and is advantageous in describing multi-matching scenarios. Therefore, this paper treats the three-domain utility-optimization matching problem as a hierarchical bipartite hypergraph-matching problem. To obtain suitable matching relationships, two methods, namely the two-stage and integrated approaches, are used for matching.

#### 3.2.1. Description of the Three-Domain Model Based on Hypergraphs

Hypergraph of the service domain: The hypergraph model of the service domain is denoted as $H_{S}\left(V_{S},E_{S}\right)$, where each business $s_{k}$ is a node in the service domain hypergraph, and the nodes in the service domain are denoted as $V_{S}=S$. Business entities belonging to the same category $type_{k}^{s}$ can form a hyperedge within the service domain, and each category of business is considered a hyperedge. Thus, the set of hyperedges in the service domain is denoted as $E_{Y}=\left\{type_{1}^{s},type_{2}^{s}\ldots \right\}$.

Hypergraph of the logical domain: The hypergraph model of the logical domain is denoted as $H_{L}\left(V_{L},E_{L}\right)$, where each service function $l_{k}$ is a node in the logical domain hypergraph, and the nodes in the logical domain are denoted as $V_{L}=L$. Service functions belonging to the same service function chain $SFC_{k}^{l}$ can form a hyperedge within the logical domain, and each service function chain is considered a hyperedge. Thus, the set of hyperedges in the logical domain is denoted as $E_{L}=\left\{SFC_{1}^{l},SFC_{2}^{l}\ldots \right\}$.

Hypergraph of the physical domain: The hypergraph model of the physical domain is denoted as $H_{W}\left(V_{W},E_{W}\right)$, where each physical component $w_{k}$ is a node in the physical domain hypergraph, and the nodes in the physical domain are denoted as $V_{W}=W$. Physical components belonging to the same category $type_{k}^{w}$ can form a hyperedge within the physical domain, and each category of physical components is considered a hyperedge. Thus, the set of hyperedges in the physical domain is denoted as $E_{W}=\left\{type_{1}^{k},type_{2}^{k}\ldots \right\}$.

#### 3.2.2. Building Service Function Chains

The construction of service function chains is fundamental to meeting customized business requirements. However, due to the interdependencies among the set of service functions that form a service function chain, the exact order of service functions cannot be fully determined. Therefore, depending on different strategies, the construction approach for service function chains varies, leading to differences in service capabilities.

To address the issue of low construction efficiency caused by the unknown logical sequence of SFCs, this paper proposes an SFC construction algorithm based on an improved lexicographic order, inspired by the lexicographic-order method.

The algorithm starts with a current construction plan as a base to encode the SFs in the SFC. This encoding ensures a one-to-one correspondence between SFs and their codes in the form of key-value pairs, enabling the generation of all possible SFC construction plans in lexicographic order. Then, based on the dependencies of service requests, rapid indexing is performed to obtain all SFC groups (SFCGs) that meet the criteria. Finally, decoding is performed to obtain all eligible SFCGs. The steps of Algorithm 1 are as follows:

Step 1: When the SFCG is empty, encode the service functionalities in the business request, establishing a one-to-one correspondence using key-value pairs. For example, if the business request contains service functionalities $\left\{s_{1},s_{2},s_{3},s_{4}\right\}$, encode them using lexicographic order and store them as $\left\{s_{1}:1,s_{2}:2,s_{3}:3,s_{4}:4\right\}$.

Step 2: Perform a lexicographic permutation of the encoded service functionalities to obtain all possible service function chains.

Step 3: Based on the dependencies among service functionalities in the business request, use the encoding to rapidly filter and obtain the SFCG that meets the criteria.

Step 4: Decode the eligible construction plans to obtain the SFCG.

The process of the SFC construction algorithm based on the improved lexicographic order is outlined as follows:
**Algorithm 1: SFC Construction Algorithm Based on Improved Lexicographic Order**1:Initialize the business request and its service functionality dependencies2:for each service request do3:   Randomly select a construction plan for encoding4:   Obtain all construction sets SFCG of SFC according to the lexicographic order.5:      $\mathrm{for}\;SFC_{i}\;\in \;SFCG\prime \;$ do6:         $\mathrm{if}\;SFC_{i}\;$ does not satisfy the dependency relationship7:            
$\mathrm{del}\;SFC_{i}$
8:         end if9:      end for10:end for

#### 3.2.3. The Two-Stage Matching of the Service Domain, Logic Domain, and Physical Domain

The first stage involves matching between nodes in the service domain and hyperedges in the logic domain. For the service domain, each service desires the best service experience. Therefore, given that the business wishes to meet its requirements, it will select the service chain with the highest service level for matching. Hence, the preference order in the service domain corresponds to the order of service levels in service chains. 

For the logic domain, service chains are selected based on the requirements of the business, preferring users with higher business demand levels. Thus, the preference order in the logic domain is the order of business demand levels. To ensure business demands and resource utilization, it is desirable to match businesses with service chains that are close in demand level. Therefore, the degree of matching between businesses and service chains is adjusted, measured by utility $U\left(E,\mathrm{cost}\right)$.

The second stage involves matching between nodes in the logic domain and nodes in the physical domain. The second stage is influenced by the matching in the first stage. Since the matching probability between businesses and service chains and the matching probability of services are determined, determining the matching between services and physical components only requires that the resources of physical components satisfy the needs of the services. The available physical nodes can be considered as the set of physical components on the shortest path between the starting and ending points of the business. Considering load balancing, the number of resources consumed by each component in the set is proportional to the ratio of its resources to the total resources of the available physical component set multiplied by the resources needed by the service chain.

#### 3.2.4. The Integrated Matching of the Service Domain, Logic Domain, and Physical Domain

The difference between integrated matching and two-stage matching lies in the approach to selecting physical components after choosing a service function chain for a business. In integrated matching, if the total resources available in the current set of usable physical components cannot meet the resource requirements of the service function chain, the current service function chain becomes unavailable. It needs to be excluded from the service-function-chain group, and the matching probability weights of the service functions need to be reoptimized.

The integrated matching approach involves selecting service function chains and physical components together, treating service functions and physical components as a unified entity. This approach eliminates scenarios where suitable service function chains exist but appropriate physical components are unavailable, thereby improving the service rate for businesses.

Below is the matching algorithm based on utility optimization (Algorithm 2):
**Algorithm 2: Optimization Matching Algorithm for Business-SFC-Physical Component Based on Utility**1:Initialize the business requirements and their corresponding service function chain groups with service levels.2:Generate preference lists for each business and service function chain.3:Place all unmatched businesses into UNMATCH4:while UNMATCH≠∅5:  
for i∈UNMATCH
6:  The requester sends matching requests to all service function chains in the list that meet its requirements7:      for j∈ SFCs that meet the requirements8:               Compute the matching probability to optimize utility9:      end for10:  end for11:  if the total resources of the available physical component set > resources required by the SFC12:    Match Successful 13:  else if the available physical component resources are insufficient14:    Delete the currently matched service function chain and re-execute step 6.15:  end if16:end while

#### 3.2.5. The Complexity Analysis of the Algorithms

In the two-stage matching, the first stage involves matching between the service domain and the logical domain, resulting in the relationship between business and service functions, with a time complexity of ${O(N}_{s}N_{L})$, where $N_{s}$ represents the number of businesses and $N_{L}$ represents the number of service functions. The second stage involves matching between service functions and physical components, resulting in the relationship between service functions and physical components, with a time complexity of ${O(N}_{L}N_{W})$, where $N_{L}$ represents the number of service functions and $N_{W}$ represents the number of physical components. The overall time complexity is ${O(N}_{s}\left(N_{L}+N_{W}\right))$. For integrated matching, treating service functions and physical components as a single entity, the total number of combinations is $N_{L}N_{W}$, resulting in a time complexity of ${O(N}_{s}N_{L}N_{W})$.

It can be observed that the time complexity of the two-stage matching is lower. As $N_{L},N_{W}$ increases, the advantage of the two-stage matching in terms of time becomes more significant, making it more promising for applications with time-sensitive requirements.

## 4. Simulation

The simulation experiments in this paper were conducted on a device equipped with an Intel Core I7-12700H processor and 16 GB of memory, using Matlab (version 2018b), Netlogo (version 6.3.0), and PyCharm (version 2022.2) software for simulation. In order to evaluate the proposed method (Algorithm 2), two matching methods were set up, with the method from reference [16] serving as a comparison. In the comparison matching method, for the matching between the service domain and the logic domain, each business selects a service function chain from its matched group with the same matching probability for each service function chain. In the matching between the logic domain and the physical domain, the shortest path algorithm is used to find the set of available physical nodes in the network, and services are deployed on the physical nodes with the largest available resource capacity.

**Figure 4:**
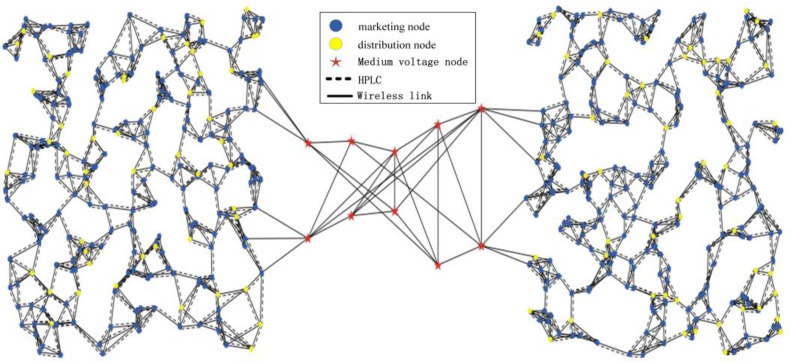
Topology of grid devices.

The simulation considers two microgrids, with a total of 500 grid devices randomly distributed. Among them, there are 400 marketing nodes responsible for generating marketing-related services and 100 distribution nodes responsible for generating distribution-related services. The links between nodes in the microgrid can be either wireless or wired. The communication radius between nodes within the microgrid is set to 50 m. The connection between the two microgrids is established through a medium-voltage power grid, distributed within an area of 900m×300m. The service data in the network topology are as shown in Table 1 and Table 2, and simulation parameters are shown in Table 3. The corresponding network topology is illustrated in Figure 4.

According to the network load, different numbers of SFC demands are dynamically generated on the grid devices. The number of service function instances in each SFC demand is randomly generated between three and six. The same type of virtual network function (VNF) is instantiated only once in each SFC. The simulation parameters are presented in the following table. To make the scenario more general, this study quantifies resource consumption using “units”.

**Figure 5:**
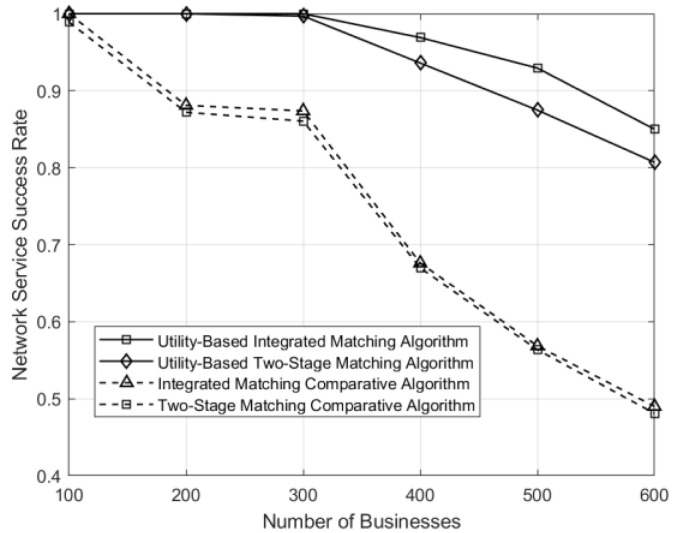
Comparison of network-service success rates under different business counts for various matching schemes.

The success rates of the business–service function chain and service function chain–physical component under different matching schemes are shown in Figure 5. Under the utility optimization-based matching approach, businesses can select the most suitable service function chain to match their needs, minimizing resource consumption while meeting demands. Therefore, the success rate is higher as the number of businesses increases. From the graph, it can be observed that both two-stage and integrated matching methods using utility optimization outperform the control matching algorithm. In two-stage matching, the selection of service function chains does not consider the resource availability in the physical domain, leading to situations where the chosen service function chain cannot be deployed on suitable physical components, resulting in a lower network-service success rate compared to the integrated approach. The success rate of two-stage matching exceeds 80%, while that of integrated matching exceeds 85%.

**Figure 6:**
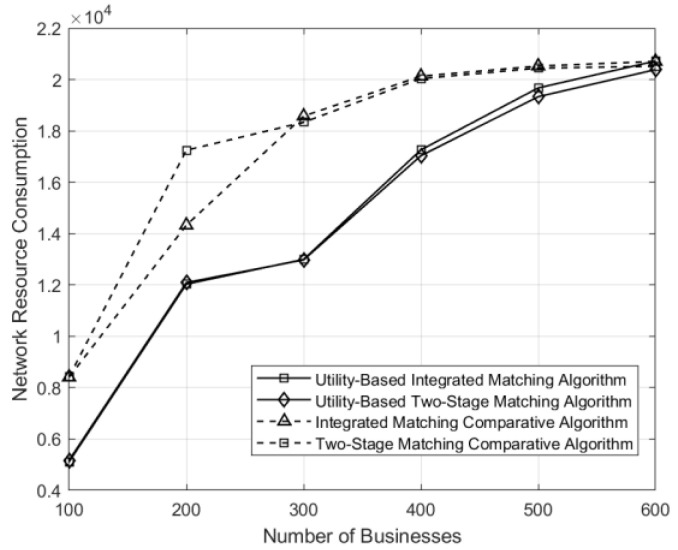
Comparison of network resource consumption under different matching schemes across varying numbers of businesses.

**Figure 7:**
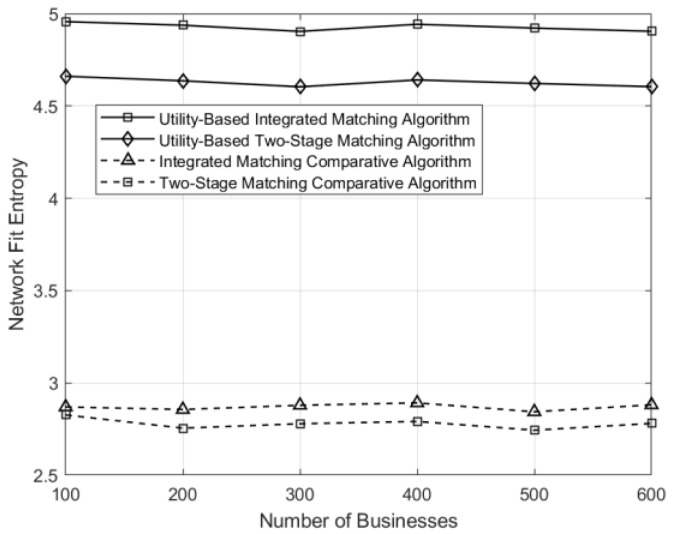
Comparison of network entropy under different matching schemes across varying numbers of businesses.

**Figure 8:**
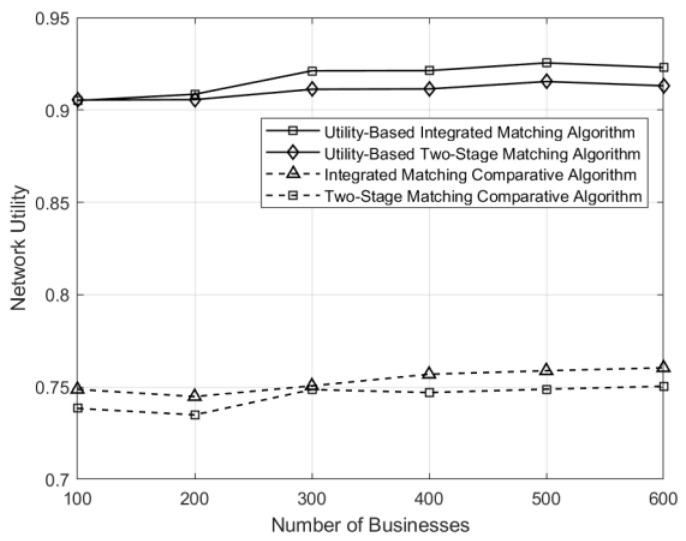
Comparison of network utility under different matching schemes across varying numbers of businesses.

The curves depicting the variations of network resource consumption, conformity entropy, and average network utility under different matching schemes in the network are illustrated in Figure 6, Figure 7 and Figure 8, respectively. Under the control matching scheme, where all service function chains within a matching group are equally likely to be selected for a given business, there is uniform probability across all service function chains. For service function chains with excessively high service levels, there exists a significant disparity between their service levels and the requirements of the business, leading to resource wastage compared to the utility-based optimization in both integrated and two-stage matching. Consequently, the utility-based optimization algorithms demonstrate significant improvements in network resource utilization and conformity compared to the control matching algorithm.

**Figure 9:**
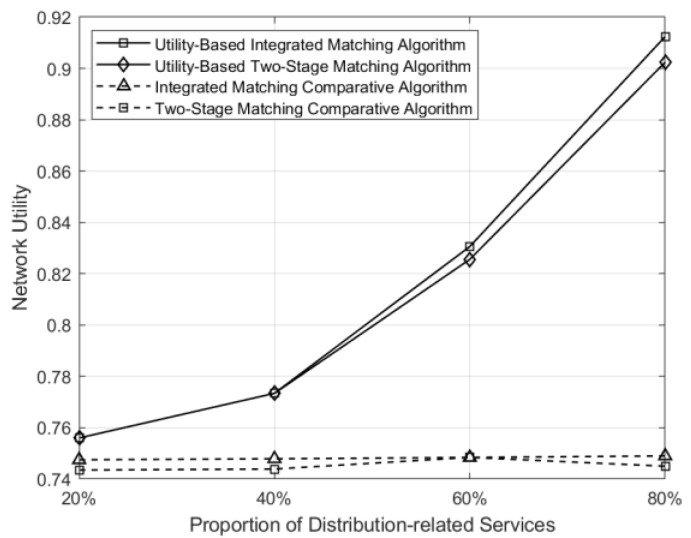
Comparison of network utility under different matching schemes across different business proportions.

The variation of utility in the network with different matching schemes as the proportion of distribution-class businesses changes is shown in Figure 9. It can be seen that when the number of businesses remains constant and the proportion of distribution-class businesses is 20%, the difference in network utility among the four schemes is not significant. This is because distribution-class businesses have higher demands compared to marketing-class businesses. When the proportion of distribution-class businesses is low, the distribution of service-level demands among various types of businesses in the marketing class is relatively uniform in the network, and the control matching scheme also adopts uniform matching. Therefore, the utility-based matching scheme does not show a significant improvement. However, as the proportion of distribution-class businesses increases, the control matching scheme still employs uniform matching, failing to adapt well to changes in business demands. In contrast, the utility optimization-based scheme can adjust the matching probabilities according to business demands, resulting in a significant improvement in network utility.

**Figure 10:**
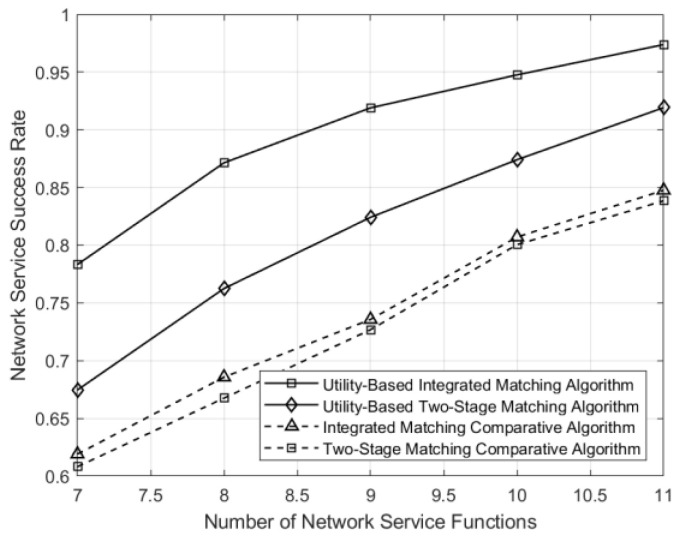
Comparison of business-service success rates across different numbers of service functions under different matching schemes.

The change in business-service success rates with the number of service functions under different matching schemes in the network is shown in Figure 10. In a fixed number of businesses in the network environment, the more service functions there are, the finer the granularity of resource allocation, leading to higher resource utilization and a finer granularity of business selection, thus improving the business-service success rate. From Figure 10, it can be observed that the business-service success rates of all four matching methods increase with the increase in the number of service functions. However, under the utility-optimization scheme, the resource utilization rate is higher, leading to a higher business-service success rate. It is worth noting that an increase in the number of service functions can lead to finer granularity, resulting in more choices for businesses. In this case, the complexity of optimization also increases. Therefore, for delay-sensitive businesses, it is important to find a balance between granularity division and optimization complexity.

## 5. Conclusions

This paper investigates assessment and optimization algorithms for the alignment between services and resources in the communication network of the intelligent distribution network on the medium- and low-voltage sides. By establishing a three-layer matching model from the service domain to the logical domain, and then to the physical domain, this paper comprehensively considers the adaptation process of the power network to the internal business of the network and defines the alignment entropy and network utility, constructing a comprehensive evaluation system that considers network performance and cost.

Under the definition of alignment entropy, this paper proposes a comprehensive method to evaluate the degree of alignment between service function chains and businesses. By considering multiple dimensions of requirements, such as latency, reliability, and accuracy, and integrating different performance indicators through weighted summation, the evaluation of the alignment degree becomes more comprehensive and accurate. Furthermore, this paper introduces the concept of utility, considering the matching effectiveness between business and services from an economic perspective, providing a more comprehensive consideration of the matching degree between the network and business.

In the solution to the three-domain matching optimization problem, due to the excellent fit between the matching of the three domains and the hypergraph matching theory, this paper considers the three-domain utility-optimization matching problem as a hierarchical bipartite hypergraph-matching problem and proposes two matching schemes: two-stage matching and integrated matching. Through simulation experiments, this paper verifies the performance of these two matching methods, demonstrating the superiority of utility optimization over traditional matching.

In the simulation results and analysis, this paper deeply analyzes the trends of business-service success rate and network utility with the number of businesses and service functions. In networks with a large number of businesses, the utility-optimization matching method performs well in improving the business-service success rate and saving network resource consumption. In terms of the access success rate, integrated matching is superior to two-stage matching, but the optimization complexity of integrated matching is higher. Therefore, we can choose the matching method according to the changes in different time scales: for small changes on a small time scale, the two-stage matching method can be used to achieve fast adaptation, and for large changes on a large time scale, the integrated matching method can be used to optimize the overall performance.

Although our primary focus is on medium- and low-voltage smart distribution networks, the architecture design of the three-domain model is universally applicable. The model can be extended to other types of distribution networks and higher voltage levels. This is because the core of the three-domain model lies in achieving flexible matching between resources and services through the division of service, logical, and physical domains. This division is independent of specific voltage levels and network types.

## Figures and Tables

**Table 1:** Correspondence table of business requirements and requirement levels.

Delay Requirement	Delay Requirement Level	Reliability Requirement	Reliability Requirement Level	Precision Requirement
Less than 0.05 s	Level 1	Above 99.5%	Level 1	Entire area
Less than 0.3 s	Level 2	Above 99%	Level 2	$\mathrm{Hundreds}{/\mathrm{km}}^{2}$
Less than 5 s	Level 3	Above 98%	Level 3	$\mathrm{Dozens}{/\mathrm{km}}^{2}$
Less than 30 s	Level 4	Above 95%	Level 4	$\mathrm{Several}{/\mathrm{km}}^{2}$
Less than 60 s	Level 5	Above 90%	Level 5	Single device

**Table 2:** Service requirement level table.

Service Category	Service Type	Delay Requirement Level	Reliability Requirement Leve	Precision Requirement Level
Marketing	Equipment Monitoring	Level 3	Level 2	Level 2
	Automatic Secondary Remote Control	Level 4	Level 4	Level 1
	Environmental Monitoring	Level 3	Level 5	Level 2
	Billing Collection	Level 3	Level 3	Level 5
Distribution	Automatic Tertiary Remote Control	Level 1	Level 2	Level 2
	New Energy Regulation	Level 1	Level 1	Level 2
	Energy Storage Control	Level 1	Level 1	Level 1
	Load Control	Level 2	Level 3	Level 1

**Table 3:** Simulation parameters.

Network Parameters	Value
Number of network nodes	500
Proportion of marketing and distribution business	$\left[0.2,0.8\right]$
Performance indicators for VNF processing unit traffic delay	$U\left[1,5\right]$
Performance indicators for VNF processing unit traffic reliability	$U\left[1,5\right]$
Performance indicators for VNF processing unit traffic accuracy	$U\left[1,5\right]$
Consumption of computing resources per unit of traffic processed by VNF	$U\left[10,25\right]$
Consumption of storage resources per unit of traffic processed by VNF	$U\left[10,25\right]$
Consumption of spectrum resources per unit of traffic processed by VNF	$U\left[10,25\right]$
Consumption of power resources per unit of traffic processed by VNF	$U\left[10,25\right]$
Computing resources of distribution nodes	$U\left[100,200\right]$
Storage resources of distribution nodes	$U\left[100,200\right]$
Spectrum resources of distribution nodes	$U\left[100,200\right]$
Power resources of distribution nodes	$U\left[100,200\right]$
Computing resources of marketing nodes	$U\left[50,100\right]$
Storage resources of marketing nodes	$U\left[50,100\right]$
Spectrum resources of marketing nodes	$U\left[50,100\right]$
Power resources of marketing nodes	$U\left[50,100\right]$
Computing resources of medium-voltage nodes	$U\left[500,800\right]$
Storage resources of medium-voltage nodes	$U\left[500,800\right]$
Spectrum resources of medium-voltage nodes	$U\left[500,800\right]$
Power resources of medium-voltage nodes	$U\left[500,800\right]$

## Data Availability

Data are contained within the article.
